# Impact of late toxicities on quality of life for survivors of nasopharyngeal carcinoma

**DOI:** 10.1186/1471-2407-14-856

**Published:** 2014-11-21

**Authors:** Wen-Ling Tsai, Tai-Lin Huang, Kuan-Cho Liao, Hui-Ching Chuang, Yu-Tsai Lin, Tsair-Fwu Lee, Hsuan-Ying Huang, Fu-Min Fang

**Affiliations:** Department of Cosmetics and Fashion Styling, Cheng Shiu University, Kaohsiung, Taiwan; Department of Hematology and Oncology, Kaohsiung Chang Gung Memorial Hospital and Chang Gung University College of Medicine, Kaohsiung, Taiwan; Department of Radiation Oncology, Kaohsiung Chang Gung Memorial Hospital and Chang Gung University College of Medicine, Kaohsiung, Taiwan; Department of Otolaryngology, Kaohsiung Chang Gung Memorial Hospital and Chang Gung University College of Medicine, Kaohsiung, Taiwan; Department of Electronics Engineering, National Kaohsiung University of Applied Sciences, Kaohsiung, Taiwan; Department of Pathology, Kaohsiung Chang Gung Memorial Hospital and Chang Gung University College of Medicine, Kaohsiung, Taiwan

## Abstract

**Background:**

To investigate the impact of physician-assessed late toxicities on patient-reported quality of life (QoL) for nasopharyngeal carcinoma (NPC) patients with long-term survival.

**Methods:**

A cross-sectional survey of QoL and late toxicities was conducted in 242 NPC patients with disease-free survival of more than 5 years after treatment. The QoL was assessed by the European Research and Treatment of Cancer Quality of Life Questionnaire (EORTC QLQ-C30). Late toxicities including neuropathy, hearing loss, dysphagia, xerostomia, and neck fibrosis were recorded based on the criteria of Common Terminology Criteria for Adverse Events version 4.0 (CTCAE v.4.0). The general linear model multiple analysis of variance (GLM-MANOVA) was performed to predict factors associated with the QoL.

**Results:**

In the multifactor model of GLM-MANOVA, of the five late toxicities of CTCAE scales, neuropathy, hearing loss, and xerostomia were observed to be significantly associated with the overall outcome of the fifteen QLQ-C30 scales. A statistically significant trend (*p* <0.05) was observed, indicating that NPC survivors with more severe neuropathy, hearing loss or xerostomia had a worse outcome on global QoL, all five functional scales, and a variety of symptomatic scales.

**Conclusions:**

To improve QoL outcome for NPC survivors, the development of a modern radiotherapeutic technique should not only focus on reduction of the dose to the salivary glands, but also on anatomical structures that are involved in neuropathy and hearing loss.

**Electronic supplementary material:**

The online version of this article (doi:10.1186/1471-2407-14-856) contains supplementary material, which is available to authorized users.

## Background

Nasopharyngeal carcinoma (NPC) is a prevalent disease in Taiwan. With the advent of the treatment technique of radiotherapy (RT) or a combination of chemotherapy, NPC patients have a greater chance of living cancer free for an extended period of time. If the individual organ receives the radiation dose above the specific dose-tolerance limit, the so called late complications, which are usually chronic, irreversible and progressive, would be induced [1]. Conventionally, assessments of these sequelae were usually from the physicians’ point of view and measured according to physical outcome. Several systems for quantitatively scoring treatment-related toxicities have been developed and are continuously evolving. The National Cancer Institute Common Terminology Criteria for Adverse Events (CTCAE) system is one of the most widely used tools for documenting toxic effects caused by cancer treatments in clinical trials [2]. The CTCAE grading system not only takes into account adverse effects induced by RT, but also those induced by other treatment modalities such as chemotherapy or surgery.

In the past decades, quality of life (QoL) and its assessment have become increasingly important in health care. The concepts of QoL refer to patients’ own perception, and self-report of their physical, mental, and social functions, as well as other related symptoms [3]. There are now a variety of well-validated QoL instruments available for use in the field of oncology. The European Research and Treatment of Cancer Quality of Life Questionnaire (EORTC QLQ-C30) is a cancer-specific type of QoL instrument with good validation and has been widely used internationally for cancer patients [4].

Growing studies have involved the investigation of QoL for patients with head and neck cancer (HNC) treated with RT. However, only a few have touched on the impact of RT-related late toxicity on the outcome of patients’ QoL [5, 6]. In this study, we focused on NPC patients with long-term survival. We investigated the impact of the severity of late toxicities, which was graded by physicians based on CTCAE v.4.0, and on the QoL outcome, which was patient-reported by using the EORTC QLQ-C30.

## Methods

This is a cross-sectional study that adheres to STROBE guidelines for reporting observational research (Additional file 1). In total, 242 NPC patients with cancer-free survival of more than 5 years were enrolled. All of them were newly diagnosed NPC and treated at the Kaohsiung Chang Gung Memorial Hospital in Taiwan from January 1997 to December 2007; those with tumour relapse or second primary cancers were excluded. As regards the existence of selection bias, we compared the distributions of sociodemographic characteristics (including age, gender, marital status, and education level) and cancer stage between the study cohort and the other NPC survivors in the cancer registration database of the institute, but no statistically significant differences were found. The Medical Ethics and the Human Clinical Trial Committee at Chang Gung Memorial Hospital in Taiwan has approved the study (No. 103-1495B) and informed consent was obtained from all eligible patients. One hundred of the patients were treated with intensity-modulated RT (IMRT) and the others using non-IMRT, which included 2-dimensional RT (2DRT, n = 39), 3-dimensional conformal RT (3DCRT, n = 24), and 2DRT plus boost by 3DCRT (n = 79) at different time periods. The detailed procedures of these techniques have been described in our previous publication [7]. Table 1 lists the distributions of patient characteristics including age, gender, marital status, education years, cancer stage, RT technique, chemotherapy, and survival years at the point of investigation. Cancer stage was recorded according to the American Joint Cancer Committee (AJCC) staging system, published in 2002. Five items of late toxicities, including neuropathy, hearing loss, dysphagia, xerostomia, and neck fibrosis, which are routinely assessed by physicians for NPC survivors in our clinical practice, were recorded based on CTCAE v.4. The EORTC QLQ-C30 version 3.0 was used to assess the cancer-specific QoL status. The questionnaires have been tested in Taiwanese NPC patients and excellent reliability and validity were obtained [8]. EORTC QLQ-C30 incorporates a range of QoL issues that are relevant to a broad range of cancer patients and contains a global QoL scale, five functional scales (physical, role, cognitive, emotional, and social), three symptom scales (fatigue, pain, and nausea/vomiting), and six single items (dyspnoea, insomnia, appetite loss, constipation, diarrhoea, and financial difficulties). All scales pertaining to the QLQ-C30 range from 0 to 100. A higher score for global QoL or a functional scale indicates a relatively better level of global QoL or functioning, whereas a higher score for a symptom scale denotes greater severity of a symptom or problem(s) [4].

**Table 1:** **Patient characteristics (N = 242)**

Variables	No	%
Age, median (range) years	46 (17–78)	
≦40	73	30.2
41 ~ 60	154	63.6
>60	15	6.2
Gender		
Male	168	69.4
Female	74	30.6
Marital status		
With spouse	188	79.3
Without spouse	49	20.7
Education years		
≦6	62	26.2
6 ~ 12	118	49.8
>12	57	24.0
AJCC stage		
I	21	8.7
II	107	44.2
III	74	30.6
IV	40	16.5
Radiotherapy		
IMRT	100	41.3
Non-IMRT	142	58.7
Chemotherapy		
Yes	160	66.1
No	82	33.9
Survival years		
5 ~ 7	162	66.9
8 ~ 10	54	22.4
11 ~ 13	26	10.7

AJCC: American Joint of Cancer Committee published in 2002; IMRT: intensity modulated radiotherapy.

The mean scores of the QoL scales were calculated according to the EORTC QLQ scoring manual [9]. To deal with the missing data, the missing items were assumed to have values equal to the average of those items that were present for the respondents, if at least half of the items from the scale have been answered. For the missing form, the mean imputation was used to replace the missing data in each scale.To analyse the predictive variables associated with and the QoL scales, the general linear model multivariate analysis of variance (GLM-MANOVA) was performed. Those variables with p < 0.25 in the one-factor model of GLM-MANOVA were entered as independent variables into the multi-factor model (backward exclusion) [6]. Wilk’s λ was used to test the impact of each variable included in the model. In case of a significant association between a factor and all QoL scales taken together, a second ANOVA was performed to investigate the association between that prognostic factor and each QoL scale separately, with post-hoc testing using the Bonferroni method. A 10-point difference of the mean scores of QoL data between groups was considered clinically significant, and the effect sizes of the difference were further measured by calculating the Cohen’s D coefficient. Effect sizes of <0.50, 0.50–0.79, and ≥0.80 were regarded as small, moderate, and large, respectively [10].

## Results

### Outcomes of QoL and late toxicities

The calculated scores for the QLQ-C30 are shown in Table 2. The mean score for global quality of life was 56.7. The mean scores of the five functional scales ranged from 77.0 to 89.1, with physical and role functioning scoring higher than others. Fatigue, followed by insomnia and financial problems were the top three symptomatic problems. Concerning symptomatic late toxicities (≧ grade 2), the respective distributions were 32 (13.2%) in neuropathy, 123 (50.8%) in hearing loss, 98 (40.5%) in dysphagia, 135 (55.8%) in xerostomia, and 65 (26.9%) in neck fibrosis (Table 3). Among them, fifty (20.6%) survivors required a hearing aid because their activity of daily life was interfered, 6 (2.5%) survivors required tube feeding for severe difficulty when swallowing, and 20 (8.3%) survivors presented remarkable neck fibrosis, so regular rehabilitation was suggested.

**Table 2:** **Scores of EORTC QLQ-C30 for survivors of nasopharyngeal carcinoma**

Scales	Mean (SD)	Median	Range
Global quality of life	56.7 (20.7)	50.0	0.0-100.0
Physical functioning	86.6 (16.7)	93.3	0.0-100.0
Role functioning	89.1 (19.3)	100.0	0.0-100.0
Emotional functioning	78.4 (18.4)	75.0	22.2-100.0
Cognitive functioning	77.0 (18.9)	83.3	0.0-100.0
Social functioning	78.8 (23.1)	83.3	0.0-100.0
Fatigue	27.3 (21.6)	33.3	0.0-100.0
Nausea/Vomiting	5.7 (12.3)	0.0	0.0-66.7
Pain	18.7 (21.6)	16.7	0.0-100.0
Dyspnoea	12.4 (18.6)	0.0	0.0-100.0
Insomnia	24.7 (25.6)	33.3	0.0-100.0
Appetite loss	13.0 (21.0)	0.0	0.0-100.0
Constipation	17.5 (23.6)	0.0	0.0-100.0
Diarrhoea	10.9 (17.4)	0.0	0.0-66.7
Financial problems	23.2 (28.1)	16.7	0.0-100.0

EORTC QLQ-C30: European Organization for Research and Treatment of Cancer Quality of Life Questionnaire C30; SD: standard deviation.

**Table 3:** **Late toxicities for survivors of nasopharyngeal carcinoma**

Variables	N	%
Neuropathy		
0	196	81.0
1	14	5.8
2	18	7.4
3	14	5.8
Hearing loss		
0	87	36.0
1	32	13.2
2	73	30.2
3	28	11.6
4	22	9.0
Dysphagia		
0	32	13.2
1	122	46.3
2	92	38.0
3	6	2.5
Xerostomia		
0	7	2.9
1	100	41.3
2	133	55.0
3	2	0.8
Neck fibrosis		
0	100	41.3
1	77	31.8
2	45	18.6
3	20	8.3

### Variables associated with QoL

In the one-factor model of GLM-MANOVA, the association of each independent variable (including eight clinical variables and five CTCAE variables) with the dependent variables (fifteen scales of QLQ-C30) was investigated (Table 4). We observed that gender, education years, RT technique, and survival years in the clinical variables and all five of the CTCAE variables were significantly (*p* <0.05) associated with the overall outcome of QLQ-C30. In the multifactor model (backward exclusion), those variables with p < 0.25 in one-factor model were entered as independent variables; years of education, RT technique, and survival years in the clinical variables and neuropathy, hearing loss, and xerostomia in the CTCAE variables remained statistically significant.

**Table 4:** **The GLM-MANOVA model of the effects of the CTCAE scales on the fifteen EORTC QLQ-C30 scales**

EORTC QLQ-C30				
	One-factor model*		Multifactor model**	
Variable	Wilk’s λ	***P value***	Wilk’s λ	***P value***
Clinical variable				
Age (≦40 *v* 41–60 *v* >60)	0.844	0.177	0.878	NS
Gender (Male *v* Female)	0.891	0.048	0.881	NS
Education years (≦6 yrs *v* 6-12 yrs *v* >12 yrs)	0.779	0.004	0.766	0.003
Marital status (Without *v* with partner)	0.922	0.285	--	--
AJCC stage (I *v* II *v* III *v* IV)	0.765	0.088	0.876	NS
Radiotherapy technique (Non-IMRT *v* IMRT)	0.806	<0.001	0.739	0.002
Chemotherapy (No *v* Yes)	0.894	0.058	0.957	NS
Survival years (5 ~ 7 yrs *v* 7-10 yrs *v* >10 yrs)	0.795	0.012	0.795	0.021
CTCAE				
Neuropathy (0 *v* 1 *v* 2 *v* 3)	0.650	<0.001	0.686	0.001
Hearing loss (0 *v* 1 *v* 2 *v* 3 + 4)	0.658	<0.001	0.713	0.02
Dysphagia (0 *v* 1 *v* 2 *v* 3)	0.702	0.002	0.755	NS
Xerostomia (0 *v* 1 *v* 2 *v* 3)	0.631	<0.001	0.697	0.003
Neck (0 *v* 1 *v* 2 + 3)	0.782	0.006	0.853	NS

GLM-MANOVA: general linear model multivariate analysis of variance; EORTC QLQ-C30: European Organization for Research and Treatment of Cancer Quality of Life Questionnaire C30; CTCAE: Common Terminology Criteria for Adverse Events, v4.0; AJCC: American Joint of Cancer Committee published in 2002; IMRT: intensity modulated radiotherapy; NS: not significant; *The one factor model: only one independent variable was entered into the model; **The multifactor model: variables with p < 0.25 in one-factor model were entered as independent variables in the model (Backward exclusion).

### CTCAE neuropathy and QoL outcome

The major causes of the 32 cases with symptomatic (grade 2 and 3) CTCAE neuropathy were cranial neuropathy in 20 cases, including 17 hypoglossal palsy, two brachial plexopathy and one optic neuropathy, temporal lobe necrosis (n = 8), and ischaemic stroke related to carotid artery stenosis (n = 4). A statistically significant trend (*p* <0.05) was observed, indicating that NPC survivors with more severe neuropathy had a worse outcome on global QoL, all five of the functional scales, and the five symptomatic scales (fatigue, nausea/vomiting, pain, insomnia, and financial problems) (Table 5). In the case of grade 2 neuropathy, a moderate to large impact (Cohen’s D: 0.42–0.96) was observed on all scales of QLQ-C30. For the 14 cases with grade 3 neuropathy, a large effect (Cohen’s D: 0.90–1.38) was observed on global QoL, all five functional scales, and the symptomatic scales of fatigue and pain.

**Table 5:** **The relationship between the CTCAE grading of neuropathy and the scores of the individual EORTC QLQ-C30 scales and the effect size of the differences**

	CTCAE grading of neuropathy										
	Grade 0 (n = 196)	Grade 1 (n = 14)			Grade 2 (n = 18)			Grade 3 (n = 14)			
	Mean (SD)	Mean (SD)	Cohen’s D	ES	Mean (SD)	Cohen’s D	ES	Mean (SD)	Cohen’s D	ES	***p***
Global quality of life	60 (18)	54 (23)	0.31	M	41 (22)	0.95	L	33 (23)	1.26	L	<0.001
Physical functioning	89 (13)	88 (13)	0.13	S	76 (22)	0.75	M	61 (27)	1.33	L	<0.001
Role functioning	91 (16)	92 (14)	0.00	S	80 (28)	0.53	M	60 (28)	1.38	L	<0.001
Emotional functioning	81 (17)	82 (15)	0.04	S	65 (24)	0.77	M	61 (19)	1.10	L	<0.001
Cognitive functioning	79 (18)	77 (14)	0.10	S	69 (22)	0.48	M	58 (25)	0.95	L	0.006
Social functioning	82 (21)	75 (13)	0.37	M	62 (29)	0.81	L	52 (26)	1.28	L	<0.001
Fatigue	25 (20)	25 (16)	0.01	S	42 (27)	0.70	M	46 (21)	1.02	L	0.001
Nausea/Vomiting	5 (11)	4 (7)	0.12	S	10 (14)	0.42	M	15 (20)	0.65	M	0.008
Pain	16 (20)	17 (20)	0.04	S	37 (24)	0.96	L	37 (26)	0.90	L	<0.001
Dyspnoea	11 (17)	8 (15)	0.22	S	22 (26)	0.47	M	21 (25)	0.47	M	NS
Insomnia	22 (24)	23 (21)	0.04	S	41 (24)	0.76	M	40 (37)	0.58	M	0.006
Appetite loss	12 (20)	7 (14)	0.27	S	22 (26)	0.46	M	24 (33)	0.44	M	NS
Constipation	17 (23)	13 (22)	0.17	S	28 (26)	0.45	M	21 (28)	0.18	S	NS
Diarrhoea	10 (17)	6 (13)	0.31	M	19 (21)	0.44	M	14 (22)	0.21	S	NS
Financial problems	20 (26)	24 (24)	0.16	S	46 (26)	0.84	L	40 (32)	0.71	M	0.001

Abbreviations: CTCAE: Common Terminology Criteria for Adverse Events, v4.0; EORTC QLQ-C30: European Organization for Research and Treatment of Cancer Quality of Life Questionnaire C30; SD: standard deviation; Cohen’s D was calculated relative to grade 0; ES: effect size based on Cohen’s D; S: small; M: moderate; L: large; NS: not significant.

### CTCAE hearing loss and QoL outcome

A statistically significant trend (*p* <0.05) was observed, indicating that those survivors with more severe CTCAE hearing loss presented a worse outcome in global QoL, all five functional scales, and six of the symptomatic scales (fatigue, nausea/vomiting, pain, dyspnoea, appetite loss, and financial problems) (Table 6). In the case of grade 2 hearing loss, a moderate effect (Cohen’s D: 0.43–0.68) was observed on global QoL, all five functional scales, and four of the symptomatic scales of QLQ-C30. For the 50 cases with grade 3 hearing loss, a large effect (Cohen’s D: 0.81–0.94) was observed on global QoL and three functional scales (physical, role, and cognitive functioning).

**Table 6:** **The relationship between the CTCAE grading of hearing loss and the scores of the individual EORTC QLQ-C30 scales and the effect size of the differences**

	CTCAE grading of hearing loss										
	Grade 0 (n = 87)	Grade 1 (n = 32)			Grade 2 (n = 73)			Grade 3-4 (n = 50)			
	Mean (SD)	Mean (SD)	Cohen’s D	ES	Mean (SD)	Cohen’s D	ES	Mean (SD)	Cohen’s D	ES	***p***
Global quality of life	62 (20)	58 (18)	0.20	S	54 (21)	0.68	M	49 (21)	0.94	L	<0.001
Physical functioning	91 (13)	91 (13)	0.04	S	86 (18)	0.55	M	77 (19)	0.92	L	<0.001
Role functioning	92 (19)	92 (15)	0.02	S	90 (18)	0.52	M	81 (22)	0.84	L	<0.001
Emotional functioning	84 (18)	78 (19)	0.33	S	77 (19)	0.47	M	73 (17)	0.64	M	0.005
Cognitive functioning	82 (17)	77 (19)	0.28	S	77 (17)	0.60	M	70 (22)	0.81	L	<0.001
Social functioning	85 (21)	82 (23)	0.13	S	77 (21)	0.43	M	69 (25)	0.70	M	<0.001
Fatigue	22 (19)	21 (18)	0.04	S	31 (23)	0.46	M	35 (22)	0.67	M	<0.001
Nausea/Vomiting	2 (7)	7 (13)	0.51	M	8 (14)	0.53	M	8 (16)	0.48	M	0.007
Pain	15 (22)	13 (17)	0.11	S	22 (24)	0.32	S	25(19)	0.53	M	0.007
Dyspnoea	8 (14)	14 (17)	0.38	S	16 (23)	0.47	M	14 (19)	0.39	S	0.022
Insomnia	21 (25)	21 (26)	0.01	S	29 (27)	0.33	S	28 (24)	0.30	S	NS
Appetite loss	8 (14)	11 (16)	0.25	S	16 (22)	0.44	M	19 (29)	0.51	M	0.009
Constipation	16 (20)	11 (16)	0.27	S	17 (24)	0.03	S	24 (31)	0.20	S	NS
Diarrhoea	9 (16)	11 (18)	0.13	S	13 (17)	0.26	S	24 (31)	0.21	S	NS
Financial problems	19 (27)	21 (25)	0.08	S	22 (23)	0.14	S	12 (19)	0.49	M	0.020

Abbreviations: CTCAE: Common Terminology Criteria for Adverse Events, v4.0; EORTC QLQ-C30: European Organization for Research and Treatment of Cancer Quality of Life Questionnaire C30; SD: standard deviation; Cohen’s D was calculated relative to grade 0; ES: effect size based on Cohen’s D; S: small; M: moderate; L: large; NS: not significant.

### CTCAE xerostomia and QoL outcome

A statistically significant trend (*p* <0.05) was also observed, which revealed that those survivors with more severe CTCAE xerostomia presented a worse outcome in all of the QLQ-C30 scales (Table 7). In the 133 cases with grade 2 xerostomia, a moderate to severe effect (Cohen’s D: 0.57–1.48) was observed on global QoL, all five functional scales, and five of the symptomatic scales of QLQ-C30. There were only two cases with grade 3 xerostomia; however, a large effect (Cohen’s D: 1.63–5.76) was observed in 14 scales (except insomnia) of QLQ-C30.

**Table 7:** **The relationship between the CTCAE grading of xerostomia and the scores of the individual EORTC QLQ-C30 scales and the effect size of the differences**

	CTCAE grading of xerostomia										
	Grade 0 (n = 77)	Grade 1 (n = 100)			Grade 2 (n = 133)			Grade 3 (n = 2)			
	Mean (SD)	Mean (SD)	Cohen’s D	ES	Mean (SD)	Cohen’s D	ES	Mean (SD)	Cohen’s D	ES	***p***
Global quality of life	77 (12)	63 (18)	0.97	M	51 (22)	1.48	L	42 (12)	2.94	L	<0.001
Physical functioning	98 (5)	91 (11)	0.79	M	83 (19)	1.10	L	70 (5)	5.76	L	<0.001
Role functioning	95 (13)	95 (11)	0.02	S	84 (23)	0.58	M	67 (0)	3.21	L	<0.001
Emotional functioning	82 (15)	86 (15)	0.28	S	73 (18)	0.57	M	50 (24)	1.63	L	<0.001
Cognitive functioning	83 (17)	83 (17)	0.02	S	73 (19)	0.59	M	50 (24)	1.63	L	<0.001
Social functioning	95 (13)	87 (17)	0.52	M	72 (25)	1.20	L	50 (24)	2.39	L	<0.001
Fatigue	11 (16)	19 (186)	0.49	M	34 (22)	1.18	L	56 (16)	2.83	L	<0.001
Nausea/Vomiting	2 (5)	3 (7)	0.51	M	10 (15)	0.77	M	33 (0)	2.65	L	<0.001
Pain	5 (13)	9 (15)	0.33	S	26 (23)	1.14	L	50 (24)	2.39	L	<0.001
Dyspnoea	10 (16)	8 (14)	0.12	S	16 (21)	0.34	S	33 (0)	2.08	L	0.008
Insomnia	29 (30)	18 (21)	0.42	M	30 (27)	0.03	S	50 (24)	0.79	M	0.002
Appetite loss	5 (13)	7 (14)	0.19	S	17 (23)	0.64	M	83 (24)	4.16	L	<0.001
Constipation	14 (18)	14 (20)	0.12	S	19 (25)	0.23	S	83 (24)	3.31	L	0.032
Diarrhoea	5 (13)	7 (15)	0.19	S	13 (18)	0.54	M	50 (24)	2.39	L	0.003
Financial problems	5 (13)	17 (24)	0.65	M	28 (30)	1.03	L	50 (24)	2.39	L	0.002

Abbreviations: CTCAE: Common Terminology Criteria for Adverse Events, v4.0; EORTC QLQ-C30: European Organization for Research and Treatment of Cancer Quality of Life Questionnaire C30; SD: standard deviation; Cohen’s D was calculated relative to grade 0; ES: effect size based on Cohen’s D; S: small; M: moderate; L: large.

## Discussion

The primary endpoint in the current study is to answer what radiation-induced late toxicities assessed by physicians significantly affect the patient-reported QoL outcome for NPC patients with long term survival. The physician-rated quantitatively scoring morbidity systems such as the Danish Head and Neck Cancer Group (DAHANCA) and Radiation Therapy Oncology Group (RTOG) systems have been found to be significantly correlated with general QoL domains of the EORTC QLQ-C30 in HNC patients [6, 11]. As far as we know, our study is the first to use the CTCAE system to investigate the association of late morbidity outcome with patient’s QoL. Results of the multivariate analysis indicated that neuropathy, hearing loss, and xerostomia of CTCAE morbidity scales had a statistically significant and clinically relevant impact on the general QoL domains of QLQ-C30 for NPC survivors.

Radiation-induced neuropathy is a chronic handicap, usually appearing several years after RT. Tissue fibrosis/necrosis or vessel occlusion may play an important role [12]. The major causes of neuropathy in our cases included cranial neuropathy, followed by temporal lobe necrosis, and carotid artery stenosis. The occurrence of cranial neuropathy for NPC patients increases with improved long-term survival. In our cohort, 13.2% presented with symptomatic neuropathy, and in the study by Kong et al., the cumulative incidences of cranial neuropathy were as high as 10.4%, 22.4%, 35.5%, and 44.5% at 5, 10, 15, and 20 years, respectively [13]. As it is progressive and often irreversible, radiation-induced neuropathy is usually a frightening development for patients. However, as far as we know, the impact of neuropathy on the QoL for NPC survivors has seldom been explored in the literature. Our study highlighted the significance of radiation-induced neuropathy in association with the QoL outcome for NPC survivors, revealing that the greater the severity of neuropathy measured by physicians, the worse the outcome of broad aspects of QoL reported by patients.

Radiation-induced otitis media can cause conductive deafness, presenting with ear stuffiness, tinnitus, and hearing loss. Hearing loss may be transient and begin as early as 3 months after the completion of RT, but it can also become chronic and progressive and last for a lifetime [14]. In our patients, the frequency of hearing loss was 50.4%, second only to xerostomia. Despite the common incidence, radiation-induced hearing loss is usually difficult to treat, and the current methods are not always effective. Many studies have demonstrated that hearing problems, such as chronic otitis media, tinnitus, or hearing loss, significantly deteriorated the physical or mental QoL status of adolescents or elderly adults in the general population [15, 16]. As expected, hearing loss was a devastating problem for NPC survivors and like neuropathy the severity of hearing loss had a significantly negative impact on QoL domains.

Xerostomia rather than dysphagia was observed to have a more pronounced impact on the overall QoL outcome in our study. This result is in contrast to the report by Lovell et al. [17]. In their study, they used the University of Washington Quality-of-Life Questionnaire and the Swallow Quality-of-Life Questionnaire to investigate the impact of dysphagia on the QoL for NPC survivors. Of the 51 cases who responded, 43 (84%) had self-reported dysphagia and those with dysphagia reported a significantly lower QoL. Dysphagia is usually multifactorial and strongly associated with xerostomia and it is difficult for assessors to judge whether xerostomia or dysphagia would impact more on patients’ QoL. In the CTCE v.4.0, it is not possible to differentiate the distinct difference between dysphagia and xerostomia, e.g. grade 2 dysphagia “symptomatic and altered eating/swallowing”, which is similar to grade 2 xerostomia “oral intake alteration, e.g. diets limited purees and/soft, moist foods”. Therefore, in clinical practice, many patients were regarded simultaneously with the same severity of dysphagia and xerostomia. Meanwhile, both grade 3 dysphagia and xerostomia in CTCAE v.4.0 are defined as “tube feeding is indicated”. We believe that low grade dysphagia might be xerostomia-related in most cases and high grade can be attributed to the dysfunction of swallowing structures. As a result, we regarded the six cases with tube feeding and tongue atrophy due to hypoglossal palsy as grade 3 dysphagia and the other two cases with tube feeding but without tongue atrophy as grade 3 xerostomia.

Some reports have shown that radiation-induced dysphagia in HNC plays an important role in QoL domains and have highlighted the importance of not only parotid sparing by modern IMRT techniques, but also preserving the pharyngeal muscles that are involved in swallowing function during irradiation [18–20]. However, in the report by Teguh et al., they observed that dysphagia was tumour site-specific, and that NPC patients suffered from less dysphagia than oropharyngeal cancer patients did [18]. We found that, in contrast to other anatomic sites of HNC, NPC survivors presented some specific but common late sequelae related to the irradiation field, such as otitis media, hypothalamic-pituitary-thyroid dysfunction, and neuropathy related from temporal lobe necrosis, cranial nerve palsy, or carotid arterial stenosis, etc. [21–23]. Besides parotid sparing for the prevention of xerostomia or dysphagia, the modern conformal radiation technique should place more emphasis on the anatomic structures that are involved in these late complications, e.g. cochlea, thyroid and pituitary gland, temporal lobe, and carotid artery. Furthermore, regular examinations such as carotid duplex scanning or evaluation of thyroid function for early detection and possibly intervention of these potential late complications should be kept in mind in routine clinical practice especially for those with high risk factors and long term survival [22, 23].

This study has several limitations. First, no pre-treatment QoL data were available in this cross-sectional study and the post-treatment late toxicities assessed by physicians were subjective. It was difficult to determine whether the late toxicities after treatment were the result of treatment or the result of the pre-existing cancer. Also, about two thirds of our patients were treated with a combination of chemotherapy, and we could not exclude the morbidity being related to chemotherapy. Second, only surviving patients receiving regular follow-up were enrolled, which might have caused selection bias. Third, the study cohort included the evolved heterogeneous radiotherapeutic components from 2D, 3D conformal to IMRT techniques at different time periods and the dosimetric data were not provided in the cohort; therefore, it was difficult to establish the specific variables of the RT technique and survival years that might have confounded the analysis.

## Conclusions

To improve QoL outcome for NPC survivors, the development of a modern radiotherapeutic technique should not only focus on reduction of the dose to the salivary glands, but also on anatomical structures that are involved in neuropathy and hearing loss.

## Electronic supplementary material

Additional file 1: **STROBE statement—checklist of items that should be included in reports of observational studies.** (DOCX 44 KB)
