# Supplementary material for: Impact of late toxicities on quality of life for survivors of nasopharyngeal carcinoma
Source: BMC Cancer. 2014 Nov 21;14:856. doi: 10.1186/1471-2407-14-856 (PMC4247772; doi:10.1186/1471-2407-14-856)
Supplement: Supplementary file 1 — Additional file 1: STROBE statement—checklist of items that should be included in reports of observational studies. (DOCX 44 KB) [file 12885_2014_5040_MOESM1_ESM.docx]

STROBE Statement—checklist of items that should be included in reports of observational studies

|  | Item No. | Recommendation | Page  No. | Relevant text from manuscript |
| --- | --- | --- | --- | --- |
| **Title and abstract** | 1 | (*a*) Indicate the study’s design with a commonly used term in the title or the abstract | p.1 | Impact of late toxicities on quality of life for survivors of nasopharyngeal carcinoma |
|  |  | (*b*) Provide in the abstract an informative and balanced summary of what was done and what was found | p.2, 3 | of the five late toxicities of CTCAE scales, neuropathy, hearing loss, and xerostomia were observed to be significantly associated with the overall outcome of the fifteen QLQ-C30 scales. A statistically significant trend (*p*< 0.05) was observed, indicating that NPC survivors with more severe neuropathy, hearing loss or xerostomia had a worse outcome on global QoL, all five functional scales, and a variety of symptomatic scales. |
| Introduction | | | |  |
| Background/rationale | 2 | Explain the scientific background and rationale for the investigation being reported | p.4 | Growing studies have involved the investigation of QoL for patients with head and neck cancer (HNC) treated with RT. However, only a few have touched on the impact of RT-related late toxicity on the outcome of patients’ QoL… |
| Objectives | 3 | State specific objectives, including any prespecified hypotheses | p. 4 | We investigated the impact of the severity of late toxicities, which was graded by physicians based on CTCAE v.4.0, and on the QoL outcome, which was patient-reported by using the EORTC QLQ-C30. |
| Methods | | | |  |
| Study design | 4 | Present key elements of study design early in the paper | p. 4 | This is a cross-sectional study… |
| Setting | 5 | Describe the setting, locations, and relevant dates, including periods of recruitment, exposure, follow-up, and data collection | p.4 | In total, 242 NPC patients with cancer-free survival of more than 5 years were enrolled. All of them were newly diagnosed NPC and treated at the Kaohsiung Chang Gung Memorial Hospital in Taiwan from January 1997 to December 2007…. |
| Participants | 6 | (*a*) *Cohort study*—Give the eligibility criteria, and the sources and methods of selection of participants. Describe methods of follow-up  *Case-control study*—Give the eligibility criteria, and the sources and methods of case ascertainment and control selection. Give the rationale for the choice of cases and controls  *Cross-sectional study*—Give the eligibility criteria, and the sources and methods of selection of participants | p.4 | All of them were newly diagnosed NPC and treated at the Kaohsiung Chang Gung Memorial Hospital in Taiwan from January 1997 to December 2007; those with tumour relapse or second primary cancers were excluded….. |
|  |  | (*b*) *Cohort study*—For matched studies, give matching criteria and number of exposed and unexposed  *Case-control study*—For matched studies, give matching criteria and the number of controls per case | nil |  |
| Variables | 7 | Clearly define all outcomes, exposures, predictors, potential confounders, and effect modifiers. Give diagnostic criteria, if applicable | p.5 | Table 1 lists the distributions of patient characteristics including age, gender, marital status, education years, cancer stage, RT technique, chemotherapy, and survival years at the point of investigation….. |
| Data sources/ measurement | 8* | For each variable of interest, give sources of data and details of methods of assessment (measurement). Describe comparability of assessment methods if there is more than one group | nil |  |
| Bias | 9 | Describe any efforts to address potential sources of bias | p.4 | As regards the existence of selection bias, we compared the distributions of sociodemographic characteristics (including age, gender, marital status, and education level) and cancer stage between the study patients and the other NPC survivors in the cancer registration database of the institute, but no statistically significant differences were found. |
| Study size | 10 | Explain how the study size was arrived at | nil |  |

Continued on next page

| Quantitative variables | 11 | Explain how quantitative variables were handled in the analyses. If applicable, describe which groupings were chosen and why | nil |  |
| --- | --- | --- | --- | --- |
| Statistical methods | 12 | (*a*) Describe all statistical methods, including those used to control for confounding | p. 5-6 | To analyse the predictive variables associated with and the QoL scales, the general linear model multiple analysis of variance (GLM-MANOVA) was performed….. |
|  |  | (*b*) Describe any methods used to examine subgroups and interactions | nil |  |
|  |  | (*c*) Explain how missing data were addressed | p.5 | To deal with the missing data, the missing items were assumed to have values equal to the average of those items that were present for the respondents, if at least half of the items from the scale have been answered. For the missing form, the mean imputation was used to replace the missing data in each scale. |
|  |  | (*d*) *Cohort study*—If applicable, explain how loss to follow-up was addressed  *Case-control study*—If applicable, explain how matching of cases and controls was addressed  *Cross-sectional study*—If applicable, describe analytical methods taking account of sampling strategy | nil |  |
|  |  | (*e*) Describe any sensitivity analyses | nil |  |
| Results | | | | |
| Participants | 13* | (a) Report numbers of individuals at each stage of study—eg numbers potentially eligible, examined for eligibility, confirmed eligible, included in the study, completing follow-up, and analysed | nil |  |
|  |  | (b) Give reasons for non-participation at each stage | nil |  |
|  |  | (c) Consider use of a flow diagram | nil |  |
| Descriptive data | 14* | (a) Give characteristics of study participants (eg demographic, clinical, social) and information on exposures and potential confounders | p.5 | Table 1 lists the distributions of patient characteristics including age, gender, marital status, education years, cancer… |
|  |  | (b) Indicate number of participants with missing data for each variable of interest | nil |  |
|  |  | (c) *Cohort study*—Summarise follow-up time (eg, average and total amount) | nil |  |
| Outcome data | 15* | *Cohort study*—Report numbers of outcome events or summary measures over time | nil |  |
|  |  | *Case-control study—*Report numbers in each exposure category, or summary measures of exposure | nil |  |
|  |  | *Cross-sectional study—*Report numbers of outcome events or summary measures | p.6-7 | The calculated scores for the QLQ-C30 are shown in Table 2. The mean score for global quality of life was 56.7… |
| Main results | 16 | (*a*) Give unadjusted estimates and, if applicable, confounder-adjusted estimates and their precision (eg, 95% confidence interval). Make clear which confounders were adjusted for and why they were included | p.7 | We observed that gender, education years, RT technique, and survival years in the clinical variables and all five of the CTCAE variables were significantly (*p* < 0.05) associated… |
|  |  | (*b*) Report category boundaries when continuous variables were categorized | nil |  |
|  |  | (*c*) If relevant, consider translating estimates of relative risk into absolute risk for a meaningful time period | nil |  |

Continued on next page

| Other analyses | 17 | Report other analyses done—eg analyses of subgroups and interactions, and sensitivity analyses | nil |  |
| --- | --- | --- | --- | --- |
| Discussion | | | | |
| Key results | 18 | Summarise key results with reference to study objectives | p.8-9 | Results of the multivariate analysis indicated that neuropathy, hearing loss, and xerostomia of CTCAE morbidity scales had a statistically significant and clinically relevant impact on the general QoL domains of QLQ-C30 for NPC survivors. |
| Limitations | 19 | Discuss limitations of the study, taking into account sources of potential bias or imprecision. Discuss both direction and magnitude of any potential bias | p.11-12 | This study has several limitations. First, no pre-treatment QoL data were available in… |
| Interpretation | 20 | Give a cautious overall interpretation of results considering objectives, limitations, multiplicity of analyses, results from similar studies, and other relevant evidence | p.11 | Some reports have shown that radiation-induced dysphagia in HNC plays an important role in QoL domains and have highlighted the importance of not only.. |
| Generalisability | 21 | Discuss the generalisability (external validity) of the study results | p.9 | In our cohort, 13.2% presented with symptomatic neuropathy, and in the study by Kong et al., the cumulative incidences of cranial neuropathy were as high as 10.4%, 22.4%, 35.5%, and 44.5% at 5, 10, 15, and 20 years, respectively… |
| Other information | |  | | |
| Funding | 22 | Give the source of funding and the role of the funders for the present study and, if applicable, for the original study on which the present article is based | nil |  |

*Give information separately for cases and controls in case-control studies and, if applicable, for exposed and unexposed groups in cohort and cross-sectional studies.

**Note:** An Explanation and Elaboration article discusses each checklist item and gives methodological background and published examples of transparent reporting. The STROBE checklist is best used in conjunction with this article (freely available on the Web sites of PLoS Medicine at http://www.plosmedicine.org/, Annals of Internal Medicine at http://www.annals.org/, and Epidemiology at http://www.epidem.com/). Information on the STROBE Initiative is available at www.strobe-statement.org.
